# THE Effect of Mentoring Programmes on Newly Graduated Nurses' Retention and Turnover: An Umbrella Review

**DOI:** 10.1111/jan.70326

**Published:** 2025-10-27

**Authors:** Elina Södergård, Jonna Juntunen, Heli‐Maria Kuivila, Marco Tomietto, Kristina Mikkonen

**Affiliations:** ^1^ Research Unit of Health Science and Technology University of Oulu Oulu Finland; ^2^ Department of Nursing, Midwifery and Health, Faculty of Health and Life Sciences Northumbria University Newcastle upon Tyne UK

**Keywords:** mentoring, mentoring programme, newly graduated nurse, retention, transition programme, turnover

## Abstract

**Aim:**

To summarise the effect of mentoring within mentoring programmes on the retention and turnover of newly graduated nurses in healthcare settings.

**Design:**

An umbrella review.

**Methods:**

Two independent reviewers screened the titles, abstracts and full texts for eligibility and critically appraised the included reviews using the JBI critical appraisal. The findings were tabulated and synthesised.

**Data Sources:**

The search was conducted in five electronic databases (CINAHL, OvidMedline, ProQuest, Scopus, Cochrane and Medic) in November 2023.

**Results:**

Out of 450 Papers, 13 systematic and integrative reviews were included. Thirteen mentoring programmes were identified and categorised into three groups based on their content: didactic mentoring programmes, interaction‐based mentoring programmes and combined mentoring programmes. Across these programme types, retention among newly graduated nurses ranged from 72% to 100% at the 1‐year mark and 70% to 98% at 2 years. Turnover rates showed consistent reductions, with post‐intervention rates ranging from 3.5% to 20% compared to pre‐intervention rates of up to 50%. Several studies reported statistically significant improvements in retention and turnover, particularly in programmes integrating structured education and preceptorship models.

**Conclusions:**

Several different mentoring programmes have been developed to support the transition of newly graduated nurses. Mentoring programmes that provide ongoing support and structured guidance increase retention and reduce turnover among newly graduated nurses.

**Implications for the Profession and/or Patient Care:**

Effective mentoring programmes are key to ensuring high‐quality patient care and a sufficient supply of qualified nurses in the future.

**Impact:**

The findings can provide information for developing transition support and mentoring programmes for newly graduated nurses.

**Reporting Method:**

This umbrella review adhered to the Preferred Reporting Items for Systematic Reviews and Meta‐Analyses (PRISMA) statement.

**Patient or Public Contribution:**

No patient or public contribution.

**Trial and Protocol Registration:**

The umbrella review protocol was registered in PROSPERO: CRD42023478044.

**Summary**

- What does this paper contribute to the wider global clinical community?
  ○ This paper strengthens the evidence that various mentoring programmes improve retention and reduce turnover of newly graduated nurses, which may help address the ongoing nursing crisis.
  ○ There is no conclusive evidence regarding the most effective mentoring programme as the duration and content vary widely. However, the ongoing support during these programmes emerges as a critical factor for the retention of newly graduated students.
  ○ While the immediate benefits of these programmes are evident, their long‐term impact remains unclear. Future research should prioritise longitudinal studies to rigorously evaluate the sustained effects of mentoring on nurse retention and turnover.

## 1 Introduction

One of the main workforce challenges facing healthcare systems worldwide is the shortage of nurses. The World Health Organization (WHO) has estimated that the total number of nurse graduates needs to be increased by 8% every year, which matches the improved capacity of their employment and, above all, the retention of graduates. Even with these changes, the shortage of nurses will be 5.7 million by 2030 (WHO 2020). Studies have identified a concerning trend of newly graduated nurses leaving the profession within their first year (Ulupinar and Aydogan 2021; Van Camp and Chappy 2017), underscoring the significance of retention initiatives. Van Patten and Bartone (2019) argue that nurses are not educated to fulfill broad professional responsibilities. After graduation, they move from a supervised and safe environment to working independently and making decisions under highly stressful conditions (Abdelaziz et al. 2020; Van Patten and Bartone 2019). Such working circumstances reduce retention (Eckerson 2018; Van Camp and Chappy 2017), and poor work engagement directly impacts the current and future nursing workforce (Voss et al. 2022).

Newly graduated nurses are a vital resource for health services, so retaining them in healthcare organisations is crucial during the current workforce crisis. Healthcare organisations have implemented structured programmes to retain newly graduated nurses (Horner 2020), including mentoring elements to support belonging, acceptance, professional development and commitment to work (Wakefield et al. 2023; Labrague and De Ios Santos 2020). However, there is limited evidence regarding the effectiveness of those strategies in reducing turnover and improving retention among newly graduated nurses (Walker and Norris 2020).

This umbrella review summarises previous research on the association of mentoring or precepting within mentoring programmes (transition programmes) with newly graduated nurses' turnover and retention in healthcare settings. From the perspective of evidence‐based nursing and healthcare organisations, this study could provide critical information for developing mentoring programmes, thereby potentially improving retention and reducing turnover of newly graduated nurses.

## 2 Background

Today's workplace demands rapid development of competence in clinical qualifications and critical thinking (Fordham 2021), which is particularly challenging for first‐year graduate nurses. The combination of limited work experience and the ongoing changes and growing complexity within the healthcare sector often leads newly graduated nurses to contemplate changing positions or even leaving the profession (Lindfors et al. 2022). Newly graduated nurses have found it beneficial to get support from a resource person they can consult, such as a mentor or a preceptor (Van Patten and Bartone 2019), and structured orientation programs can help strengthen new nurses' professional identity and commitment to a career in healthcare (Lindfors et al. 2022).

A mentor is an experienced practitioner who establishes a long‐term, trusting relationship with a newly graduated nurse (Voss et al. 2022). Mentoring can occur both within and outside the clinical setting and aims to support the newly graduated nurse's personal and professional growth. This relationship typically spans more years (Smith and Jones 2020). Conversely, a preceptor is an experienced nurse assigned to orient a newly graduated nurse to their role and work environment (Pohjamies et al. 2022). This relationship is typically short‐term, lasting weeks to a few months, and is confined to the clinical setting during work hours (Key and Wright 2017). Both the mentor's and preceptor's roles include teaching, guiding and supporting the newly graduated nurses as they acclimate to patient care and the complexities of the nursing profession (Smith and Jones 2020; Voss et al. 2022). Mentorship, however, involves wider coaching, role modelling, advocating and sharing both personal and career advice (Keogh 2015).

In this umbrella review, mentoring is defined as the structured support and guidance provided by an experienced nurse to a newly graduated nurse to develop clinical skills, professional competence and self‐confidence and facilitate the transition into the nursing profession and community. The mentor serves multiple roles—as a guide, supervisor and educator, supporting the novice nurse's growth into a competent practitioner. Preceptorship is regarded as a form of mentoring, and this review includes all programmes in which newly graduated nurses are formally assigned a mentor or preceptor. Only formal mentoring is included in this review, defined as relationships established through a planned process, typically initiated by management in a top‐down manner (Keogh 2015). Informal mentoring, characterised by voluntary, peer‐initiated interactions without a structured framework, is excluded.

Various programmes have been developed around the world to promote transition and professional development (Eckerson 2018), and they have a common aim to provide support to newly graduated nurses in their transition to the workplace and improve retention (Graf et al. 2020). There is wide variation in the duration and structure of the programmes (Phillips et al. 2017). However, it is suggested that a structured transition programme that lasts more than 4 months has been shown to provide satisfaction from preceptees and facilitate the transition into the workplace better than unstructured programmes (Walker and Norris 2020).

Mentoring can improve newly graduated nurses' retention (Eckerson 2018; Horner 2020; Zhang et al. 2019), and successful transition decreases workforce turnover (Pohjamies et al. 2022). Programmes that include mentoring lead to positive experiences, help build self‐confidence, support professional development (Van Camp and Chappy 2017; Van Patten and Bartone 2019; Voss et al. 2022), and can contribute to a sense of belonging (Horner 2020). Mentoring enhances the work engagement of newly graduated nurses (Eckerson 2018; Horner 2020; Zhang et al. 2019), and a smooth transition helps reduce turnover (Pohjamies et al. 2022).

In this umbrella review, we use the retention and turnover rates to indicate the engagement of newly graduated nurses at work. The term turnover could be linked with retention, and these terms are often used interchangeably, but their meanings and measurements differ. In general, turnover means someone leaving their job, and turnover rates indicate the percentage of nurses leaving and being replaced in the field (Kovner et al. 2014). Retention rates refer to the percentage of nurses remaining in their current position or field at the end of a defined period (Lee et al. 2024). Organisations aim to achieve high staff retention and low nursing staff turnover rates. Our umbrella review exclusively focused on actual turnover or retention outcomes without accounting for subjective factors such as intent to stay or leave, nurse disengagement or levels of professional commitment. Retention and turnover rates are often used as outcome measures for the effectiveness of mentoring programmes (Cochran 2017).

## 3 Aim

The aim of this umbrella review was to determine the effect of mentoring within mentoring programmes on newly graduated nurses' retention and turnover rates in healthcare settings.

## 4 Methods

### 4.1 Design

This study was conducted as an umbrella review using the Joanna Briggs Institute Manual for Evidence Synthesis. An umbrella review was performed to establish evidence from existing systematic reviews, as this method is designed to cover a wider array of scholarly subjects and associated issues (Aromataris et al. 2020). This umbrella review provides a thorough overview of previous research on the association of mentoring programmes with newly graduated nurses' retention and turnover rates in healthcare settings. The umbrella review was reported following the preferred reporting items for systematic reviews and meta‐analyses (Page et al. 2021) and was registered with PROSPERO (NIHR, n.d.) under the identifier [blinded].

### 4.2 Search Methods and Eligibility Criteria

The search strategy developed aims to select systematic and integrative reviews and meta‐analyses published on the subject. An information specialist assisted in developing the search strategy, which includes MeSH terms and keywords and is combined with Boolean operators AND or OR for each included electronic database. Electronic databases CINAHL, OvidMedline, ProQuest, Scopus, Cochrane and Medic were searched between 31 October and 2 November 2023. The detailed search strategy for each database can be found in Data S1. Time limitations were not set, and the English, Finnish or Swedish publications were considered.

The PCC framework (participants, concept and context) (Aromataris et al. 2020) was used to define the inclusion criteria for systematic reviews. Participants included newly graduated nurses with a professional qualification in nursing. Newly graduated midwives and paramedics were also included in the search strategy. In many countries, these professions are recognised as equivalent in scope of practice and educational preparation to nursing (WHO 2020), and within the European Union legislative framework, they hold qualifications that permit registration as nurses (European Parliament and Council 2005; European Council 2013). Including these professions broadens the evidence base by incorporating insights from comparable transition‐to‐practice experiences in other health disciplines, where newly qualified practitioners navigate similar clinical organisational and emotional challenges. Moreover, given the increasing emphasis on interprofessional team‐based care, examining mentoring in midwifery and paramedicine enriches the understanding of mentoring approaches relevant to nursing practice and supports the development of more adaptable and inclusive mentoring models.

In this umbrella review, we included studies that defined newly graduated nurses as those within their first 3 years of clinical practice. While many systems define the first 12 months as the formal orientation period, our decision to include up to 3 years was based on the inclusion ranges used in several of the included reviews. In this umbrella review, we included studies that defined newly graduated nurses as those within their first 3 years of clinical practice. While many systems define the first 12 months as the formal orientation period, our decision to extend the timeframe to 3 years was informed by the inclusion ranges used in several of the included reviews and underpinned by Benner's novice‐to‐expert model (Benner et al. 2009). According to Benner, nurses typically progress from Novice to Advanced Beginner within the first year, gaining basic situational awareness but still requiring substantial guidance. The stage of Competent, often reached after approximately 2 to 3 years of practice, is characterised by deliberate planning, prioritisation and the ability to work more independently, although flexibility and adaptive responses to complex or rapidly changing situations are still developing. In contrast, the Proficient stage, where practitioners demonstrate intuitive grasp and adaptive mastery, generally occurs beyond this period. By including up to 3 years of practice, we aimed to capture literature on sustained support strategies during the critical developmental stages from Advanced Beginner to Competent, which are particularly relevant to enhancing retention, reducing turnover and fostering readiness for autonomous, confident practice. If a review also included competent or experienced nurses beyond this timeframe, results were only extracted when they were presented separately for newly graduated nurses.

Additionally, this umbrella review focused on structured mentoring programmes and/or transitional programmes incorporating mentoring or preceptorship, with reported outcomes related to retention and turnover. The context was limited to healthcare settings. Full inclusion and exclusion criteria are outlined in Table 1.

**TABLE 1:** Inclusion and exclusion criteria, PCC format.

PCC	Inclusion criteria	Exclusion criteria
Participants (P)	Nurses with professional qualifications in nursing. Nurses who have graduated for up to 3 years.	Other health care professionals than nurses (e.g., doctors, physiotherapists) Nurses who have been registered for more than 3 years (e.g., competent or experienced nurses) Nurses who are not yet registered (e.g., students).
Concept (C)	Mentoring programmes, preceptorship programmes or mentoring programmes occur during the transition phase or in the early stage of the newly graduated nurses' careers. Mentoring could be included in other transition programmes, for example, Nurse Residency Programme Actual retention, Actual turnover.	Other transitional programmes that do not include mentoring or preceptorship elements. Informal mentoring. Other point of view than actual retention or turnover, for example, work satisfaction without the aspect of work engagement and intention to stay or leave.
Context (C)	All health care environments where nursing occurs, and where newly graduated nurses work.	Non‐health care settings. Studies that do not focus on the context of nursing (e.g., from the perspective of management).
Type of study	Systematic literature reviews and meta‐analyses (e.g., systematic reviews, meta‐analysis, meta‐synthesis, narrative review, integrative review). Language of the study: English, Finnish or Swedish.	Other reviews and study designs (e.g., comprehensive or scoping reviews, qualitative or quantitative studies). Language of the study other than English, Finnish or Swedish.

### 4.3 Search Outcome

**FIGURE 1:**
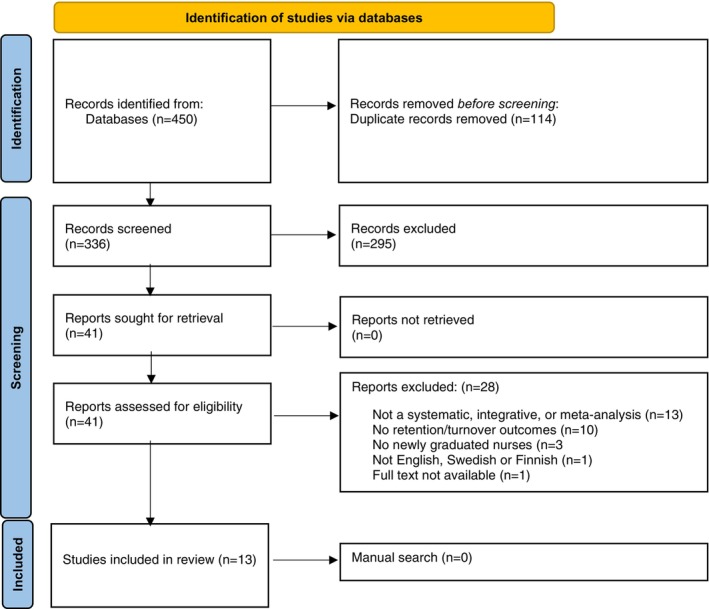
PRISMA flow diagram (Page et al. 2021).

A total of 450 publications were retrieved from the database searches and uploaded into the Covidence Systematic Review Software tool (2021). Initially, 114 duplicate studies were eliminated, leaving 336 studies screened. Each study underwent a double screening process. The first two researchers (ES and JJ or H‐MK) read the titles and abstracts, and then the full texts, individually and based on the inclusion criteria. A third reviewer resolved any disagreements. Based on titles and abstracts, 295 studies were eliminated. After full‐text screening of 41 studies, 28 studies did not meet the initial inclusion criteria and were eliminated. Thirteen articles met the inclusion criteria and were subjected to extraction and quality appraisal. A manual search of reference lists from these papers was conducted to ensure all relevant studies were included in the umbrella review, but no additional studies were found. The PRISMA flow diagram is presented in Figure 1.

### 4.4 Quality Appraisal

All included studies were critically appraised for methodological quality by two independent reviewers (ES and JJ) using the standard JBI Critical Appraisal Checklist for Systematic Reviews and Research Synthesis (Aromataris et al. 2020). The assessment tool examines a research article's reliability, quality and results across 11 different aspects, which were analysed using the criteria yes, no, unclear or not applicable. The quality assessment was largely consensual, and all disagreements were discussed and resolved. All studies were included in the umbrella review, regardless of their quality score. The quality assessment scores were predominantly high, with 62% of reviews scoring between 9 and 11 points, and 38% scoring between 6 and 8 out of 11 points. The lowest scores were achieved in question 7, which asked whether methods were used during the data extraction phase to minimise the risks of errors. The scores of methodological quality assessment for each study are shown in Data S2.

### 4.5 Data Abstraction

The included studies were extracted based on (1) authors, year, country, title, (2) study type, (3) methodology, (4) number of participants, content including duration, content and key elements of programmes, (5) context and (6) key findings including the effect of the programmes on the retention or turnover of newly graduated nurses (Tables 2 & 3). Two researchers (ES and JJ) conducted the data extraction, while the third researcher (KM) reviewed the extracted articles for any missing information to ensure that all relevant results were identified. In the event of disagreements, the authors discussed them until a consensus was reached. Data extraction focused solely on the results of each systematic review, rather than on the primary studies included in those systematic reviews. The key findings of each review were retrieved from the results to the extent that they addressed the research question.

**TABLE 2:** Extracted data of studies included for the umbrella review.

Author/s, year, country and title	Study type	Methodology	Participants	Concept	Context	Key findings
Ackerson and Stiles (2018), USA. Value of Nurse Residency Programs in Retaining New Graduate Nurses and Their Potential Effect on the Nursing Shortage	Systematic literature review	Quantitative studies (*n* = 26): descriptive designs (*n* = 21), outcome (*n* = 2), and quasi‐experimental (*n* = 1) Mixed methods (*n* = 2).	New Graduate Nurses (*n* = 12,317).	Nurse Residency Programmes nine internally developed, with 10–12 months, and evidence‐based programmes such as Versant or UHC/AANC programmes (17, 4–12 months) The value of Nurse Residency Programmes on retention or turnover rates at 1‐year and 2‐year marks.	Acute care setting.	In all included studies, the Nurse Residency Programmes improved nurses' retention or turnover rates at the 1‐year mark. The overall retention rates were from 72.5% to 100% and turnover rates from 2% to 13% at the 1‐year mark. The positive effect on retention rates stays from 1 year (90%–95%) to 2 years (70%–85%), and in turnover rates from 1 year (5%–7.1%) to 2 years (9%–19.6%). The impact of nurse residency programmes was not strong at the 2‐year mark. There was a slight difference between internally developed and established programmes. The average retention rate of internally developed programmes was 2.77% less than that of established Nurse residency programmes. The most crucial factor in program effectiveness is the content of the program rather than its theoretical framework.
Asber (2019), USA. Retention Outcomes of New Graduate Nurse Residency Programs	Integrative Review	Quantitative studies (*n* = 16): Study designs not reported.	New graduate registered nurse (amount not reported).	Nurse residency programmes, which included eight established programmes such as UHC/AANC or Versant and six organisation‐based programmes or one program with a mixed structure Length from 3 to 12 months. The effect of Nurse Residency Programmes on retention or turnover rates after 1 year of hire.	Hospital setting.	All programmes improved the retention of newly graduated nurses. The retention rates were higher than the national average in the 1‐ and 2‐year marks. Programme structure may impact retention. Three of the lowest retention rates were in organisation‐based programmes, and the highest was in formally defined and established Nurse Residency Programmes (UHC/AACN or Versant). In established programmes, the 1‐year retention rates ranged from 90.6% to 100% (the UHC/AACN was 90.6%–100%, and the Versant NRP was 92.9%–93.6%). In organisation‐based programmes, the rates ranged from 74% to 98%. Overall, 2‐year retention rates (78.8%–91%) were significantly higher than the national average of 51.8%.
Brook et al. (2019), UK. Characteristics of Successful Interventions to Reduce Turnover and Increase Retention of Early Career Nurses: A Systematic Review	Systematic review	Quantitative studies, interventional studies (*n* = 53): Time series nonequivalent control group *n* = 7, Time series *n* = 15, Pretest, posttest *n* = 13, Nonequivalent control group pretest posttest *n* = 7, nonequivalent control group posttest only *n* = 8, Quasi‐experimental pretest posttest *n* = 1, Nonequivalent pretest posttest non control group *n* = 1, Randomised controlled multi‐site *n* = 1.	Early career nurses, including newly graduated nurses and newly licenced/qualified nurses (*n* = 9890 and above).	Nurse residency programmes (6, 3–12 months), externship combined with NRP or internship (2, 6–12 months), internship (3, 12 months), transition to practise programmes (3, 4–12 months), preceptorship (2, 3–6 months), mentoring programme (1, 78 months), orientation programme (1, 18 months) and single externship (1, 10 weeks). To identify the benefits achieved by the interventions regarding retention and turnover.	Hospital setting.	Nurse residency programmes: The post‐intervention turnover decreased 5%–37% at the one‐year mark (compared to pre‐intervention). The turnover rates decreased 4%–29.4% 2 years post‐intervention (compared to pre‐intervention). Externship combined with NRP or internship: 14%–21% improvement in retention at 2 years post‐intervention. Internship: 8%–17.2% decrease in turnover pre‐ to post‐intervention at 1‐year mark. Transition to practice: 6%–22.5% increase in retention rates between pre‐ and post‐intervention. Preceptorship/Mentoring programme: 9%–28% decrease in post‐intervention turnover. The mentoring programme also increased retention by 13%. Orientation programme: 12.4%–28% decrease in turnover from pre‐ to post‐intervention Single externship: Turnover was 8.8% higher in the intervention group vs. Control group.
Chen and Lou (2022), Taiwan. The Effectiveness and Application of Mentorship Programmes for Recently Registered Nurses: A Systematic Review	Systematic review	Quantitative, quasi‐experimental studies (*n* = 5).	Recently registered nurses (*n* = 335).	One‐to‐one mentorship programmes (2, 3–12 months) The application of mentorship programmes for recently registered nurses.	The context is not mentioned.	Mentorship programmes with one regular, trained mentor significantly reduced the turnover rate of recently registered nurses.
Edwards et al. (2011), UK. The Effectiveness of Strategies and Interventions to Assist the Transition from Student to Newly Qualified Nurse	Systematic review	Quantitative studies (*n* = 33): RCT *n* = 1, Quasi‐experimental *n* = 3, Observational/descriptive studies *n* = 29.	Newly qualified nurses (*n* = 10,787 and above).	Interventions to assist transition include Nurse internship (3, 2–12 months), residency programmes (8, 6–12 months), graduate nurse/orientation programmes (5, 2–5 months) and Preceptorship (2, 2–6 months). The effectiveness of support strategies on newly qualified nurses' retention or turnover rates.	Clinical area.	Internship: The 1‐year turnover rate was 12% and the retention rates were 73%–74%. There was also a retention rate of 88%, but no time point was reported. Residency Programmes: The 1‐year retention rates were 78%–94% and the 2‐year rates varied from 83%–98%. A retention rate of 74% at the 4‐year mark was reported. Turnover rates were 8%–16.5% (no time point), and significant decreases were reported. There was a decrease in turnover when comparing pre‐ and post‐implementation rates. Pre‐turnover rates were 27% at the 1‐year mark and post 7,1%. At the 2‐year mark, the pre‐rate was 49% and the post‐turnover rate was 19.6%. Post implementation turnover rates were also measured in the 3‐year mark (28.6%), 4‐year mark (34.2%) and 5‐year mark (39.8%). Graduate nurse/orientation programmes: 1‐year retention rates were 77%–94.5%. Significant differences were noted between the intervention and comparison groups in 3, 9 and 12 months. RR 1‐year 78.8% intervention, 53.4% control (*p* = 0.015), RR 78%–82% post programme, 18 months RR 89% Preceptorship programme: No significant difference in retention rates between the programme participants and the control group. Orientation and preceptorship: The number of newly graduated nurses leaving the organisation during the first 12 months of employment reduced from 24% to 1% in 2 years since the programme was implemented. Overall, retention rates are high in every intervention, and significant differences were reported between intervention and comparison groups at 12 months in residency and graduate orientation programmes. These programmes also included a didactic element (e.g., classroom sessions and skills lab).
Edwards et al. (2015), UK. A Systematic Review of the Effectiveness of Strategies and Interventions to Improve the Transition From Student to Newly Qualified Nurse	Systematic review	Quantitative studies (*n* = 30): Quasi‐experimental *n* = 3, Comparative *n* = 5, Longitudinal *n* = 8, Case study *n* = 8, Cross‐sectional *n* = 3, RCT *n* = 1, mixed method *n* = 1, Descriptive correlation *n* = 1.	Newly qualified nurses (*n* = 9597 and above).	Residency programmes (9, 6–12 months), internship (3, 2–12 months), preceptorship programmes (2, no mention of duration) and graduate nurse orientation programmes (7, 1–20 weeks) The impact of support strategies and programmes on individual and organisational outcomes among retention.	Clinical area.	Residency programmes: Both 1‐year and 2‐year retention rates were high. 1‐year rates were 78%–94% and 2‐year rates were 83%–98%. Turnover rates were reported to be 7.1% at the 1‐year mark and 19.6% & at the 2‐ and 3‐year mark, which were lower than before programme implementation. Also, a significantly lower turnover rate was reported at 6 months (*p* < 0.05), but not in the 1‐year mark (*p* = 0.20). There were also turnover rates of 8%–16.5% with no time point reported. Internship: Three studies considered internships. One reported a turnover rate of 12% at the 1‐year mark, which is lower than in the control group (20%). The other reported a retention rate of 74%. The third study noted significantly higher retention (*p* = 0.014) and lower turnover (*p* = 0.009) in the intervention group than in the controls. Graduate nurse: 1‐year retention rates were 77%–94.5% after the programme. 18–19 months 89%–96% Preceptorship: No significant difference in retention rates between the programme participants and the control group. Orientation programme and preceptorship: Two years after the programme was implemented, the 1‐year turnover rate decreased from 24% to 1%. Overall, all strategies lead to improved or high 1‐year retention (73%–94.5%) or turnover rates (1%–16.5%), but no evidence indicates the most effective programme.
Ke et al. (2017), Taiwan. The Effects of Nursing Preceptorship on New Nurses' Competence, Professional Socialisation, Job Satisfaction and Retention: A Systematic Review	Systematic review	Quantitative studies (*n* = 6): RCT *n* = 1, Quasi‐experimental *n* = 1, Observational studies *n* = 4.	New nurses (number not reported).	Preceptorship programmes (2.3–4.5 months) The effect of the programmes on new nurses' retention.	Clinical area	The mean retention rate was 78% in the first year, and the second‐year retention rate was estimated to be 88.46%. However, little evidence supports the promotion of one‐to‐one preceptorship.
Rush et al. (2013), Canada, Best Practices of Formal New Graduate Nurse Transition Programs: An Integrative Review	Integrative review	Quantitative and qualitative studies (*n* = 47): Descriptive designs *n* = 27, quasi‐experimental *n* = 8, longitudinal *n* = 7 and qualitative *n* = 5.	New nurse graduates (*n* = 2727 and above).	Residency programmes (8, 12–15 months), un‐named programmes (6, 3–24) and mentorship (3, 12–18 months). The impact of formal transition programmes on new graduate nurses' retention and turnover.	Acute care settings.	The impact of transition programmes on new graduate nurses' retention was well‐supported. The average retention rate was 90.1%, and the average turnover rate was 10.5%. Residency programmes: The initial retention rate was 60%, and the turnover rate was 50%. After the programme, the retention rate increased to 90%, while the turnover rate decreased to 13%. The difference in retention rates was 30%, and in turnover rates, it was 37%. Overall, post‐programme retention rates ranged from 78% to 90%, and turnover rates ranged from 8% to 13%. Un‐named programmes: The pre‐programme retention rates ranged from 60% to 76%, with a turnover rate of 20%. The post‐programme retention rates increased to between 89% and 99%, while the turnover rate dropped to 6%. The difference in retention rates between pre‐ and post‐programme was 23% to 39%, and the change in turnover rates was 14%. Overall, post‐programme retention rates varied from 89% to 97%. Mentorship: The pre‐programme turnover rate was 32%, and the post‐programme rate was 16%. The change between pre‐ and post‐programme turnover rates was 16%.
Rush et al. (2019), Canada. Best Practices of Formal New Graduate Transition Programs: An Integrative Review	Integrative review	Quantitative, qualitative and mixed method studies (*n* = 76): Descriptive/descriptive‐correlational designs *n* = 30, quasi‐experimental *n* = 9, longitudinal *n* = 7, true experimental *n* = 3, mixed‐methods *n* = 12 and qualitative *n* = 15.	New graduate nurses (*n* = 3996 and above).	Residency programmes (8, 12–15 months), un‐named programmes (5, 3–12), other novice nurse transition programmes (3, 10–12 months), and mentorship (2, 12–18 months). The impact of formal transition programmes on new graduate nurses' retention and turnover.	Acute care settings.	The impact of transition programmes on new graduate nurse retention was well‐supported. The average retention rate was 88%, and the average turnover rate was 11%. Retention rates were higher for new graduate nurse programmes vs. controls. Residency programmes: The pre‐programme retention rate was 60%, and the post‐programme retention rate was 90%. The change between pre‐ and post‐programme retention rates was 30%. Overall, post‐programme retention rates varied from 78% to 90%. Un‐named programmes: The pre‐programme retention rates were 60%–76%, and post‐programme retention rates were 89%–99%. The changes between pre‐ and post‐programme retention rates were 13%–39%. Novice nurse transition programmes: The post‐programme retention rates were 80%–86%. Mentorship: The pre‐programme retention rate was 77%, and the post‐programme retention rate was 90%. The change between pre‐ and post‐programme retention rates was 13%. Overall, post‐retention rates were 90%–92%.
Salt et al. (2008), Canada. Increasing Retention of New Graduate Nurses. A Systematic Review of Interventions by Healthcare Organisations	Systematic Review	Quantitative (*n* = 16): one‐time experimental case study *n* = 9, one‐group pretest posttest *n* = 3, nonrandomised control group pretest‐posttest *n* = 3 and group comparison design *n* = 1.	New graduate nurses (*n* = 1180).	Structured preceptor programme (10), needs‐based orientation and/or speciality training (7), externship (2). The length varied from less than three months to 3–6 or 6–12 months. The effectiveness of retention strategies targeted at newly graduated nurses.	Healthcare Organisations.	Preceptor programme with one‐to‐one clinical guidance: 1‐year retention rates were 73%–94.5%. Rates are higher than in the literature. Needs‐based orientation/speciality training: The retention rates at 6 or 12 months were 77%–94.5%, higher than in the literature or the control group. Externship: 1‐year retention rate was 72%, and 2‐year rates were 50%–61.4%. The rates were higher in programme participants than in non‐participants in both programmes. Overall, the length of programmes impacts the retention rates of newly graduated nurses. Rates were higher in longer programmes.
Vázquez‐Calatayud and Eseverri‐Azcoiti (2023), Spain. Retention of Newly Graduated Registered Nurses in the Hospital Setting: A Systematic Review	Systematic review	Quantitative studies (*n* = 7): Quasi‐experimental *n* = 6 and cohort study *n* = 1.	Newly graduated registered nurses (*n* = 2158 and above).	Combined programmes with both residency programme and one‐to‐one mentoring component (3), structured residency programmes (2), individualised mentoring programmes (1) and diversified adaptive education (1). Lengths from 3 to 24 months, but 12 months was the most frequent. The effectiveness of interventions in promoting retention and/or turnover rates among newly graduated nurses.	Hospital setting.	Nurse residency and mentoring programme: The 1‐year turnover rate was 10.5%–12%, and the retention rate was 83%–88%. After 2 years, the turnover rate was 31%, and the retention rate was 77%–85%. New graduates' turnover rates were significantly reduced in the intervention group (3.5%–3.77%) compared to the control group (14%–14.07%). Programmes included core and specific competencies, as well as precepting or mentoring. Combined programmes: The turnover rate in the intervention group (3.87%) significantly decreased compared to the control group (5.06%). The mean retention rates also improved after these kinds of programmes, which included core and specific competencies and precepting or mentoring. Diversified adaptive education: The turnover rate was 12.6% over 3 months, and retention was 87.9% over 1 year. Overall, all programmes improved newly graduated nurses' turnover or retention rates, and most of them significantly. 1‐year retention rates were 83%–97.2%. It is unclear which programme is the most effective, but a 1‐year‐long nurse residency or individualised mentoring programmes seem promising.
Vidal and Olley (2021), Australia. Systematic Literature Review of Clinical Mentoring on Graduate Registered Nurses' Clinical Performance, Job Satisfaction and Retention.	Systematic literature review	Qualitative studies (*n* = 2), quantitative (*n* = 3): Study designs not reported Mixed method (*n* = 2).	New graduate registered nurses (amount not reported).	Transitional practice programmes (4), Nurse residency programmes (2) and generational mentorship (1) Lengths 1–6 years The effect of mentoring on new graduate registered nurses' retention.	The context is not mentioned.	The programmes mainly contribute to job retention, decrease turnover and address nursing shortages. Mentoring was not always clearly established to be associated with turnover.
Zhang et al. (2016), China. The Effectiveness and Implementation of Mentoring Program for Newly Graduated Nurses: A Systematic Review	Systematic Review	Quantitative sutdies (*n* = 9): Quasi‐experimental studies *n* = 8 and RCT *n* = 1.	Newly graduated nurses (*n* = 1040).	Mentoring programmes (4, 1–3 years) The effectiveness of mentoring programmes regarding turnover data.	The context is not mentioned.	The mentoring programme decreased the turnover rates of newly graduated nurses. The rates in the mentoring group were 7%–20%, and in the non‐mentoring groups, they were 20%–31%.

**TABLE 3:** Calculation of the overlap measures.

Reviews	13
Primary studies	172
Number of included publications (including double counting)	335
Covered area (CA)	0.1498
Corrected covered area (CCA)	0.0790 (7.9%)

### 4.6 Synthesis

The mentoring programmes were categorised based on the similarity of their content. Retention and turnover rates were extracted from the mentoring programmes and tabulated to facilitate reporting of the results. Finally, two authors (ES and JJ) prepared a narrative synthesis. Due to the heterogeneity and characteristics of the included reviews, meta‐analysis was not performed (Tufanaru et al. 2024).

A total of 172 primary studies were included in the 13 reviews and incorporated into this umbrella review. As an umbrella review could include systematic reviews considering the same primary studies, it is essential to calculate the overlap between the systematic reviews. For this reason, the calculated corrected area (CCA) was calculated. In detail, the overlap is categorised into slight (0%–5%), moderate (6%–10%), high (11%–15%) or very high (> 15%) (Pieper et al. 2014). This measure represents an overall estimation of the overlap between the primary studies included in the systematic reviews considered in the umbrella review.

## 5 Results

### 5.1 Characteristics of the Included Reviews

The included reviews were published between 2008 and 2023, of which 10 were systematic reviews and three were integrative reviews. The results of this umbrella review consist of over 54,000 newly graduated nurses. In most studies, participants had < 1 year of nursing experience or the mentoring programme was used within 1 year of graduation.

The reviews included in this umbrella review contained 13 different types of programmes. The structure and length of the review programmes varied from 1 week to 24 months. Retention or turnover rates were included mostly at 1‐ or 2‐year marks or in both.

Based on the content of the programmes, this umbrella review's results were categorised into three themes to describe the main features of the programmes. These categories were named as (1) mentoring programmes with didactics, (2) mentoring programmes based on interaction, and (3) combined mentoring programmes. *Mentoring programmes with didactic* included clinical support from a mentor, preceptor or another resource person. The key feature of the programmes was also the educational activities they contained. The added didactic elements meant participants had guided learning sessions such as classroom time, case studies and simulations during the programme. These programmes aimed to strengthen their skills and bridge the gap between theory and practice. The defining factor in *mentoring programmes based on interaction* is the common element that newly graduated nurses can work alongside an experienced nurse during the programme. Participants enhanced their nursing skills by working with more experienced nurses without classroom‐based teaching. *Combined mentoring programmes* were combinations of two different types of interventions, which meant that the participants went through a programme that included didactics and an interaction‐based mentoring programme. The effects of these three types of mentoring programmes are presented below. The retention outcomes are presented in Table 4, and the turnover outcomes in Table 5.

**TABLE 4:** Retention outcomes.

Included reviews	Mentoring programmes with didactics			Mentoring programmes based on interaction			Combined mentoring programmes	
	Nurse residency programme	Internship	Orientation/Transition	Preceptorship	Mentoring	Externship	Externship and nurse residence programme or internship	Residency and mentoring
Ackerson and Stiles (2018)	72.5%–100% (1‐year) 90%–95% (1‐year) to 70%–85% (2‐year) Control group: 80% Intervention group: 88.9% (*p* = 0.014)							
Asber (2019)	90.6%–100% (1‐year) 78.8%–91% (2‐year)							
Brook et al. (2019)			6%–22.5% increased between pre to post		Post: increased 13%		Post: 14%–21% improved (2‐year)	
Edwards et al. (2011)	78%–94% (1‐year) 83%–98% (2‐year)	73%–74% (1‐year) 88% (post, no reported time point)	77%–94.5% (1‐year) Control group: 53.4% Intervention group: 78.8% (*p* = 0.015) RR 78%–82% post programme (no reported time point)		No significant difference between the intervention and control group			
Edwards et al. (2015)	78%–94% (1‐year) 83%–98% (2‐year)	74% (post, no reported time point) Significantly higher in intervention group than control group (*p* = 0.014)	Post 77%–94.5% (1‐year)	Improved and unchanged results	Improved and unchanged results			
Ke et al. (2017)				78% (1‐year) 88.46% (2‐year estimated)				
Rush et al. (2013)	Pre 60% Post 60% Post 78%–90%**				Pre 60%–76% Post 89%–99% Post 89%–97% **			
Rush et al. (2019)	Pre 60% Post 60% Post 78%–90%**		Post 80%–86%		Pre 60%–77%, Post 89%–99% Post 90%–92%**			
Salt et al. (2008)			77%–94.5% (½‐1‐year)	73%–94.5% (1‐year)		72% (1‐year) 50%–61.4% (2‐year)		
Vázquez‐Calatayud and Eseverri‐Azcoiti (2023)	83%–88% (1‐year) 77%–85% (2‐year)							Post RR% improved Control: 96.1% Intervention: 97.2%
Zhang et al. (2016)					Interventions 7%–20% versus Controls 20%–31%			

Abbreviation: RR, Retention rate.

** In all included studies.

**TABLE 5:** Turnover outcomes.

Included reviews	Mentoring programmes with didactics			Mentoring programmes based on interaction			Combined mentoring programmes	
	Nurse Residency Programme	Internship	Orientation/transition	Preceptorship	Mentoring	Externship	Externship and NRP or internship	Residency and mentoring
Ackerson and Stiles (2018)	2%–13% (1‐year) 5%–7.1% (1‐year) to 9%–19.6% (2‐year) Pre 27%–50% Post 7.1%–13% (1‐year) Pre 12%–49% Post 7%–28.6% (2‐year) Pre 36.8% Post 6.41% (no time point)							
Brook et al. (2019)	Post decreased 5%–37% (1‐year) & 4%–29.4% (2‐year)	Post decreased 8%–17.2% (1‐year)	12.4%–28% decrease from pre to post	Post decreased 9%–28%		9% higher in the intervention group versus the control group		
Chen and Lou (2022)					Significant decrease of TO			
Edwards et al. (2011)	8%–16.5% (post, no reported time point) Pre 27% Post 7.1% (1‐year) Pre 49% Post 19.6% (2‐year) Post 28.6% (3‐year) Post 34.2% (4‐year) Post 39,8% (5‐year) Significant decrease reported	12% (1‐year)			Decrease from 24% to 1% (1‐year)			
Edwards et al. (2015)	8%–16.5% (post, no reported time point) 7.1% (1‐year) 19.6% (2‐year & 3‐year) Significantly lower at 6 months (*p* < 0.05), but not in the 1‐year mark (*p* = 0.20)	Intervention group: 12% Control group: 20% (1‐year) Significantly lower in intervention group than control group (*p* = 0.009)						
Rush et al. (2013)	Pre 50% Post 13% Post 8%–13% (no reported time point)				Pre 20%–32% Post 6%–16%			
Vázquez‐Calatayud and Eseverri‐Azcoiti (2023)	Control 14% versus Intervention 3.5% (*p* < 0.001) 10.5%–12% (1‐year) 31% (2‐year), change 12% (OR: 1.1, 95% CI: 0.6–2.0)		12.6% (3 months)		Control 14.07% versus Intervention 3.77% (*p* < 0.001)			Control 5.06% Intervention 3.87% (=significant decrease)
Vidal and Olley (2021)					The influence is not consistently clear			

### 5.2 Mentoring Programmes With Didactics

Three different programmes were categorised as mentoring programmes with didactics. These programmes were named as (1) Nurse Residency Programmes (NRP) or residency programmes, (2) internship programmes and (3) transition programmes. All programmes included structured teaching, such as simulations, case studies and medication calculations, along with clinical support, but the model varied from mentoring by preceptor to preceptorship. Providing peer support and opportunities for newly graduated nurses to meet and discuss their experiences was also a common theme. The common goal is to support newly graduated nurses' self‐confidence and develop the clinical skills needed to prepare them for practice so that they can provide competent and safe patient care. The programmes aim to integrate newly graduated nurses into the healthcare team, improve job satisfaction and reduce turnover. The length of the programmes varied widely from 1 week to 15 months. Internship and residency programmes were implemented in collaboration between academia and practice and were generally longer than the transition programmes.

#### 5.2.1 Nurse Residency Programmes

NRP or residency programmes were the most reported programmes in previous reviews (Ackerson and Stiles 2018; Asber 2019; Brook et al. 2019; Edwards et al. 2011; Edwards et al. 2015; Rush et al. 2013; Rush et al. 2019; Vázquez‐Calatayud and Eseverri‐Azcoiti 2023; Vidal and Olley 2021). These programmes were based on a theoretical framework such as Benner's novice to expert or Duchsher's transition theory. The NRPs could be either established, such as the University Health System Consortium/American Association of Colleges of Nursing Program (UHC/AACN), Versant NRP or Wisconsin NRP (Ackerson and Stiles 2018; Asber 2019; Edwards et al. 2011, 2015). Two reviews (Ackerson and Stiles 2018; Asber 2019) also described internally developed or organisation‐based programmes. Internally developed programmes were described based on Benner's novice to expert, Dreyfus's theory of skill acquisition, Donabedian's constructs of structure, process and outcome, Bridges' transition management, and Kolb's experiential learning cycle and stages of transition theory (Ackerson and Stiles 2018). There was no detailed description regarding the context of an organisation‐based NRP (Asber 2019).

Seven included systematic reviews showed that these programmes positively impact retention rates (Ackerson and Stiles 2018; Asber 2019; Edwards et al. 2011, 2015; Rush et al. 2013, 2019; Vázquez‐Calatayud and Eseverri‐Azcoiti 2023). At 1 year, the NRPs improved the retention rate from 73% to 100% (Ackerson and Stiles 2018; Asber 2019; Edwards et al. 2011, 2015). Rush et al. (2013, 2019) reported that post‐programme retention increased by up to 30%. Ackerson and Stiles (2018) also found a significant (*p* = 0.014) improvement in retention between the non‐participant group (80%) and the participant group (89%).

Five included reviews reported retention rates at 1‐ and 2‐year marks, with 1‐year rates ranging from 83%–100% and 2‐year rates from 77%–91% (Ackerson and Stiles 2018; Asber 2019; Edwards et al. 2011, 2015; Vázquez‐Calatayud and Eseverri‐Azcoiti 2023). Vázquez‐Calatayud and Eseverri‐Azcoiti (2023) reported retention rates of 83%–88% at the 1‐year mark and 77%–85% at the 2‐year mark (OR 1.5, 95% CI 0.9–2.5). According to Asber (2019), the retention rates were higher than national averages at the 1‐year and 2‐year marks and significantly higher at the 2‐year mark (79%–91%) than the national average of 52%. Edwards et al. (2011, 2015) noted sustained retention over time: 78%–94% at 1 year, 83%–98% at 2 years and 74% at 4 years. Despite overall positive trends, some findings indicated a decline over time. Ackerson and Stiles (2018) found a drop in retention from 90%–95% after 1 year to 70%–85% after 2 years, suggesting NRPs are more effective in the short term.

It seems that there is a small difference between internally developed or organisation‐based and established NRPs (Ackerson and Stiles 2018; Asber 2019) when the average retention rate of internally developed programmes was 3% less than in established NRPs (Ackerson and Stiles 2018). Asber (2019) also found that the lowest retention rates were in organisation‐based programmes (78%–98%), and the highest was in formally defined and established NRPs such as University Healthsystem Consortium/American Association of Colleges of Nursing Program (91%–100%) or Versant (93%–94%). So, it seems that the most critical factor in the programme's impact on retaining newly graduated nurses was the content of the programme rather than its theoretical framework (Ackerson and Stiles 2018) or the structure (Asber 2019).

A positive effect was found on newly graduated nurses' turnover rates in five included reviews (Ackerson and Stiles 2018; Brook et al. 2019; Edwards et al. 2011, 2015; Vázquez‐Calatayud and Eseverri‐Azcoiti 2023), and three included reviews reported a significant decrease in turnover when NRP was used (Edwards et al. 2011, 2015; Vázquez‐Calatayud and Eseverri‐Azcoiti 2023). Vázquez‐Calatayud and Eseverri‐Azcoiti (2023) found turnover rates of 10.5%–12% at the 1‐year mark and 31% at the 2‐year mark (OR: 1.1, 95% CI: 0.6–2.0). They reported a 12% loss of nurses between the 1‐year mark and the 2‐year mark. Ackerson and Stiles (2018) reported turnover rates from 2% to 13% at the 1‐year mark. They also noted that the positive effect on turnover rates lasts from 1 year (5%–7.1%) to 2 years (9%–19.6%), but according to them, the impact of NRPs is not strong at the 2‐year mark (Ackerson and Stiles 2018).

When comparing the turnover rates before and after the NRP programme, there was a 37% decrease in post‐programme (Rush et al. 2013). Moreover, Brook et al. (2019) found a 5%–37% decrease in turnover rates at the 1‐year mark and a 4%–29% decrease at the 2‐year mark when compared to pre‐intervention rates. Ackerson and Stiles (2018) noted decreased turnover rates when comparing pre‐ and post‐programme rates, but statistical significance was not reported. Nevertheless, Vázquez‐Calatayud and Eseverri‐Azcoiti (2023) reported that new graduated nurses' turnover rates significantly (OR: 0.2, 95% CI: 0.1–0.4, *p* < 0.001) reduced in the programme participant group (3.5%) compared to the non‐participant group (14%). Edwards et al. (2011) found a change of 20% in pre‐ to post‐turnover rates at the 1‐year mark and a 29% change at the 2‐year mark (Edwards et al. 2011). They also reported post‐implementation turnover rates at the 3‐year mark (29%), 4‐year mark (34%) and 5‐year mark (40%). Furthermore, Edwards et al. (2015) reported a significantly lower turnover rate (7%) at the 1‐year mark and 20% at the 2‐ and 3‐year marks (20%), which were lower than before programme implementation. Moreover, a significantly lower turnover rate was reported at 6 months (*p* < 0.05) but not at the 1‐year mark (*p* = 0.20) (Edwards et al. 2015).

#### 5.2.2 Internship

The internship programmes included a structured educational component related to immersion in clinical work, thereby aiming to close the gap between academic preparation and clinical practice (Edwards et al. 2011). The programme participants were assigned to a mentor or preceptor who provided clinical expertise in patient care management (Brook et al. 2019; Edwards et al. 2011, 2015). Internships were studied in three included reviews (Brook et al. 2019; Edwards et al. 2011, 2015), and two noted high retention rates (Edwards et al. 2011, 2015). Retention rates were 73%–74% at the 1‐year mark (Edwards et al. 2011) and 74%–88% with no time point reported (Edwards et al. 2011, 2015). Significantly higher retention (*p* = 0.014) was noted in the intervention group who participated in the programme than in controls who did not (Edwards et al. 2015).

Three included reviews found decreased or lower turnover rates in the intervention studies (Brook et al. 2019; Edwards et al. 2011, 2015). Brook et al. (2019) noted a decrease in turnover rates from 8%–17% between pre‐ and post‐intervention at the 1‐year mark. Edwards et al. (2011, 2015) reported a turnover rate of 12% at the 1‐year mark. This was 8% lower than in the control group that did not participate in the programme, with a significantly lower turnover rate (*p* = 0.009) in the participant group than in the non‐participant group (Edwards et al. 2015).

#### 5.2.3 Transition Programmes

The transition programmes were aimed at supporting the socialisation of newly graduated nurses into the workplace and helping them transition into the organisation (Brook et al. 2019; Edwards et al. 2011). The structure of these programmes was similar to that in nurse residency or internship programmes, featuring both classroom teaching and clinical support through one‐to‐one mentorship or preceptorship, but these programmes were generally shorter in duration (Brook et al. 2019; Edwards et al. 2011; Rush et al. 2013, 2019). Seven included reviews reported transition programmes (Brook et al. 2019; Edwards et al. 2011, 2015; Rush et al. 2019; Salt et al. 2008; Vázquez‐Calatayud and Eseverri‐Azcoiti 2023; Vidal and Olley 2021) and five reviews demonstrated positive retention outcomes (Edwards et al. 2011, 2015; Rush et al. 2019; Salt et al. 2008; Vázquez‐Calatayud and Eseverri‐Azcoiti 2023). Rush et al. (2019) reported that the post‐programme retention rates were 80%–86%. In other included systematic reviews, the 1‐year retention rates range from 77% to 95% (Edwards et al. 2011, 2015; Vázquez‐Calatayud and Eseverri‐Azcoiti 2023). Salt et al. (2008) found higher retention rates for groups that went through the programme than control groups, emphasising the efficacy of tailored educational interventions in supporting nurse retention (Salt et al. 2008). Similarly, Brook et al. (2019) found a 6%–23% increase in retention rates between pre‐ and post‐programme.

There was also a positive effect on newly graduated nurses' turnover, with five included systematic reviews showing low turnover rates (Ackerson and Stiles 2018; Asber 2019; Brook et al. 2019; Edwards et al. 2011, 2015). The 1‐year turnover rates were 2%–17% (Ackerson and Stiles 2018; Edwards et al. 2011, 2015). According to Brook et al. (2019), the turnover decreased by 12%–28% from pre‐ to post‐programme. Additionally, the Vázquez‐Calatayud and Eseverri‐Azcoiti (2023) review showed promising results, with turnover rates as low as 13% over 3 months.

### 5.3 Mentoring Programmes Based on Interaction

While many mentoring interventions share common goals—supporting newly graduated nurses during their transition to practice—some programmes focused exclusively on practice‐based and relational learning, without including structured educational components like classroom instruction or simulation. These were grouped under the theme of mentoring programmes based on interaction, as their defining feature was the emphasis on working alongside experienced nurses in real clinical settings. The support was delivered through various relational models including preceptorship (Brook et al. 2019; Edwards et al. 2011, 2015; Ke et al. 2017; Salt et al. 2008), mentorship (Brook et al. 2019; Chen and Lou 2022; Rush et al. 2013; Rush et al. 2019; Vidal and Olley 2021; Zhang et al. 2016), and externship (Brook et al. 2019; Salt et al. 2008), and learning occurred primarily through observation, guided practice and informal feedback.

These programmes varied in duration (3 to 24 months) and structure. In some cases, the distinction between mentor and preceptor roles was not clearly articulated, and mentoring styles ranged from one‐on‐one guidance to group mentoring. Below, we describe each programme type and its observed effects on retention and turnover outcomes.

#### 5.3.1 Preceptorship

Preceptorship programmes paired newly graduated nurses with experienced clinical staff in a structured but non‐didactic environment. The focus was on real‐time supervision, progressive responsibility and role‐modelling. Two included systematic reviews reported high 1‐year retention rates, ranging from 78% (Ke et al. 2017) to 95% (Salt et al. 2008). Additionally, Ke et al. (2017) noted an increase in retention from 78% at 1 year to 89% after 2 years. Positive effects on turnover were also reported: Edwards et al. (2011) found that turnover dropped from 24% to 1% over 2 years post‐implementation. Brook et al. (2019) noted a 9%–28% decrease in turnover following preceptorship programmes.

#### 5.3.2 Mentoring

Mentorship models were more varied and often included emotional and professional development support. These relationships were less formal than preceptorships and often not tied to orientation timelines. Four systematic reviews (Brook et al. 2019; Edwards et al. 2011; Rush et al. 2013, 2019) reported notable improvements in retention, including a 13%–39% increase in post‐programme retention rates (Rush et al. 2013, 2019). Retention improved from 60%–76% pre‐programme to 89%–99% post‐programme.

Regarding turnover, Rush et al. (2013) observed a 14%–16% reduction, while Zhang et al. (2019) found that mentoring groups had lower turnover (7%–20%) compared to non‐mentored groups (20%–31%). Significant reductions were also reported by Chen and Lou (2022) and Vázquez‐Calatayud and Eseverri‐Azcoiti (2023). Vázquez‐Calatayud and Eseverri‐Azcoiti (2023) reported turnover rates of 3.77% among the intervention group and 14% among the control group (*p* < 0.001). However, not all included reviews confirmed an association between mentoring and reduced turnover; Vidal and Olley (2021) and Edwards et al. (2015) found unchanged or inconsistent results.

#### 5.3.3 Externship

Externship programmes are typically offered to student nurses to provide clinical experience before graduation, often under the supervision of a preceptor. While these programmes are usually considered part of academic preparation, we included them in this umbrella review because two included systematic reviews reported post‐graduation outcomes, specifically evaluating retention and turnover among newly graduated nurses who had participated in externships during their studies (Salt et al. 2008; Brook et al. 2019).

Externship participants demonstrated improved retention outcomes in one review, with a 1‐year retention rate of 72% and 2‐year rates between 50% and 61% (Salt et al. 2008). However, Brook et al. (2019) reported higher turnover (by 9%) in programme participants compared to non‐participants, suggesting variability in effectiveness.

Although externships may be integrated within broader residency or internship structures in some settings, the included reviews treated them as distinct interventions, which justified their initial separation in our categorisation.

### 5.4 Combined Mentoring Programmes

Some included reviews reported multi‐phase or layered interventions, where newly graduated nurses participated in two or more mentoring approaches sequentially or in combination. These included, for example, an externship followed by an internship or NRPresidency programme supplemented with structured mentoring sessions or preceptorship. These interventions were categorised as combined mentoring programmes, as they integrated both didactic and interaction‐based elements within the same developmental pathway. Importantly, retention and turnover outcomes were measured after the full combined programme was completed, not separately for each component.

Brook et al. (2019) reported that integrating externships with NRPs or internships resulted in a 14%–21% improvement in retention at 2 years post‐programme. Similarly, Vázquez‐Calatayud and Eseverri‐Azcoiti (2023) found that mean retention rates improved, and turnover decreased significantly, with a 4% turnover rate for participants compared to 5% for non‐participants. Edwards et al. (2011, 2015) also reported that combining transition and preceptorship programmes resulted in a reduction of 1‐year turnover from 24% to 1% over 2 years.

## 6 Discussion

This umbrella review summarised previous research on the association of mentoring programmes with newly graduated nurses' turnover and retention in healthcare settings. Based on the findings presented, it is well supported that generally mentoring programmes improve retention and reduce turnover among newly graduated nurses, which may help address the ongoing nursing crisis. The structure and duration of the mentoring programmes vary widely, and there is no conclusive evidence regarding the most effective mentoring programme (Edwards et al. 2011, 2015; Rush et al. 2013, 2019; Salt et al. 2008; Vázquez‐Calatayud and Eseverri‐Azcoiti 2023). One review found that the length of mentoring programmes impacts retaining newly graduated nurses because the rates were higher in longer programmes (Salt et al. 2008), but other reviews did not support this.

Nevertheless, the ongoing support during these programmes emerges as a critical factor for their retention, as too little support might cause a lack of self‐confidence and decrease newly graduated nurses' retention. Van Patten and Bartone's (2019) cross‐sectional study underscored the value of supportive elements like mentorship and preceptors during this phase, highlighting their importance in any programme. However, Edwards et al. (2011) and Rush et al. (2013, 2019) found that all strategies lead to improved or high retention rates. Edwards et al. (2011) stated that the retention rates were high in every programme, and significant differences were reported between intervention and comparison groups at the 12‐month point in mentoring programmes with additional didactic elements such as NRPs, internship and transition programmes. Similarly, Wolford et al. (2019) emphasised the necessity of support for newly graduated nurses during their transition to practice and the importance of implementing an NRP to provide this support. According to the findings of this umbrella review, Vázquez‐Calatayud and Eseverri‐Azcoiti (2023) noted a significant reduction in newly graduated nurses' turnover rates between programme participants and non‐participants when using a programme that included didactics and in mentoring programmes based on interaction. In the participant groups, the turnover rate was as low as 4%, while the non‐participant groups had a turnover rate of 14% (Vázquez‐Calatayud and Eseverri‐Azcoiti 2023). These findings can be reflected by Wolford et al. (2019), who found a decrease in turnover rates among newly graduated nurses participating in NRPs compared to those who did not. Also, in a recent study by Koh et al. (2023), NRP showed an overall 2‐year attrition rate as low as 4%.

According to this umbrella review's findings, only NRPs appeared to lead to retention rates as high as 100%. These programmes fall under mentoring programmes with didactic elements, although the specific effect of the didactic components remains unclear. While programme structure varied, limited evidence suggested that content and delivery were likely key to programme success. In contrast, mentoring programmes based primarily on interaction showed more variability, including some unchanged or negative results. Despite this, mentoring programmes remain valuable and should be further developed to meet newly graduated nurses' needs, ease transition stress and support workplace integration. Importantly, attention must also be paid to the competence of mentors. Based on previous studies (Pohjamies et al. 2022; Walker and Norris 2020), mentoring should ideally be a voluntary and supportive relationship distinct from formal professional supervision. However, voluntariness alone is not sufficient; mentors must possess specific competencies to effectively guide new graduates. Identifying and strengthening these competencies is essential for successful mentoring outcomes.

The nursing shortage is a global issue that requires numerous actions to address effectively. The transition phase is demanding but also a significant time for newly graduated nurses when they gain experience and professional skills. According to Van Patten and Bartone (2019), nurses are not trained to fulfill broad professional responsibilities. Fewer clinical hours and little face‐to‐face training increase the gap between theory and practice (Graf et al. 2020), which should be bridged in collaboration between academia and clinical practice to reduce the transition shock and increase the retention of newly graduated nurses. Further development of mentoring programmes in a collaborative manner can be one factor in closing the gap. As mentioned earlier, the mentors' competence is an important factor in ensuring the success of programmes. Healthcare organisations should use academia to train mentors to fulfill the needs of newly graduated nurses.

A cross‐sectional study by Koskinen et al. (2023) indicates that successful nursing education significantly enhances job satisfaction for newly graduated nurses a year after completing their studies, underscoring the lasting impact of educational factors throughout the transition phase. Job satisfaction is related to work engagement (Yildiz and Yildiz 2022), which increases retention and reduces turnover; therefore, it could be highly beneficial to invest in nursing education. This is strengthened by the WHO (2020), which suggests that countries increase funding to educate and employ at least 5.9 million additional nurses, thereby potentially improving retention and helping address the nursing shortage. Implementing a mentoring programme also requires investment from organisations, but it may help to address the current global nursing crisis cost‐effectively. According to Zhao et al. (2019), high staff turnover increases healthcare costs. Previous reviews have shown that using a mentoring programme leads to significant cost savings (Ackerson and Stiles 2018; Brook et al. 2019), which is mainly due to the higher retention rates and lower turnover of newly graduated nurses within their first 1–3 years of employment (Brook et al. 2019). Supporting newly graduated nurses' transition mainly incurs costs related to their recruitment, educational development and support and the expenses of additional staff and replacements during support sessions (Brook et al. 2019).

Retention or turnover rates were primarily reported at either 1‐ or 2‐year marks or in both. According to Ackerson and Stiles (2018), there is a positive effect on retention rates lasting from 1 to 2 years, but the impact is not strong at the 2‐year mark. However, Asber (2019) stated that overall 2‐year retention rates were higher than the national average, suggesting an ongoing effect of the mentoring programmes. Nevertheless, it remains evident that available information regarding the influence of these programmes is limited, leaving the long‐term effects unclear; hence, it would be beneficial to focus on longitudinal research. These considerations about the long‐term impact of the mentoring programmes align with previous quantitative studies in international literature (Kim et al. 2022; Lee et al. 2024; Zhang et al. 2019.)

## 7 Limitations

The umbrella review adhered to the PRISMA standards for reporting systematic reviews, meta‐analyses and the Joanna Briggs Manual for Evidence Synthesis. However, it has several limitations that should be considered. First, only reviews published in English, Swedish and Finnish were included, which presents a risk of publication bias. Second, the methodological quality of the few reviews was rated relatively low (Data S2). This indicates that the overall rigor and reliability of the studies included in this analysis may be compromised. Third, there was a high degree of heterogeneity among the included reviews regarding the programmes utilised, their descriptions and the outcome measurements assessed. Differences in how individual reviews reported their findings can affect the synthesis of this umbrella review and introduce joint reporting bias. Some studies may present more detailed data, while others may be less specific, making it challenging to compare the results and determine the most effective mentoring programme. A limitation of this umbrella review is that descriptions of individual mentoring programs and their specific effects were constrained by the level of detail reported in the included reviews. As we synthesised data at the review level, rather than re‐examining each primary study, programme characteristics and outcomes could only be reported as presented in the source reviews. This may have limited the granularity of our analysis but ensured methodological consistency with umbrella review standards. Additionally, the findings are mostly limited to 1‐ and 2‐year marks, leaving the long‐term impact of the programmes unclear.

## 8 Conclusions

The evidence of the association of different mentoring programmes on the retention and turnover of newly graduated nurses is evident. It is highly beneficial and cost‐effective for organisations to implement a mentoring programme for newly graduated nurses. Mentoring programmes with ongoing support and guidance can increase retention and reduce turnover. There is no evidence indicating which mentoring programme is the most effective, but it is clear that newly graduated nurses need support during the challenging transition phase. The gap between theory and practice should be narrowed by investing in education and fostering good collaboration between universities and clinical facilities to provide better professional skills for newly graduated nurses. To effectively meet the needs of newly graduated nurses, the content and structure of mentoring programmes should be collaboratively developed, drawing on the combined expertise of academic institutions and clinical practitioners. Strengthening mentors' competencies is also essential to ensure the delivery of high‐quality guidance and sustained professional support throughout the programme. To this end, structured mentor training—jointly designed and implemented by academic and clinical practitioners—would be a valuable strategy to enhance the overall effectiveness of mentorship.

Retention and turnover data primarily focus on the 1‐ and 2‐year marks, consequently limiting the available evidence on the long‐term impact of mentoring programmes. More longitudinal studies and meta‐analyses of post‐programme retention and turnover rates are needed to strengthen the long‐term effects of the programmes. Further comparative studies are also needed to determine the most effective programme content.

## Author Contributions

**Elina Södergård:** study design, data collection, data analysis, manuscript writing, manuscript commenting. **Jonna Juntunen:** study design, data collection, data analysis, manuscript commenting. **Heli‐Maria Kuivila:** data collection, manuscript commenting. **Marco Tomietto:** data analysis, manuscript commenting. **Kristina Mikkonen:** study design, data collection, data analysis, manuscript commenting.

## Disclosure

There is a statistician on the author team, Kristina Mikkonen and Marco Tomietto. The authors affirm that the methods used in the data analyses are suitably applied to their data within their study design and context, and the statistical findings have been implemented and interpreted correctly. The authors agree to take responsibility for ensuring that the choice of statistical approach is appropriate and is conducted and interpreted correctly as a condition to submit to the Journal.

## Conflicts of Interest

The authors declare no conflicts of interest.

## Supporting information


**Data S1:** Search terms.

**Data S2:** Assessment of methodological quality of included studies based on JBI‐QARI (Lockwood et al., 2020).

## Data Availability

Data available on request from the authors.
