# Supplementary material for: THE Effect of Mentoring Programmes on Newly Graduated Nurses' Retention and Turnover: An Umbrella Review
Source: J Adv Nurs. 2025 Oct 27;82(7):6948–69. doi: 10.1111/jan.70326 (PMC13267445; doi:10.1111/jan.70326)
Supplement: Supplementary file 1 — Data S1: Search terms. [file JAN-82-6948-s001.docx]

| Supplementary file 1. Search Terms | | |
| --- | --- | --- |
| Database | Search strategy | Records |
| CINAHL | ( ( ("new* qualified" or "new* graduate*" or newcomer* or "early career*" or novice* or "recent* graduate*" or “recent* registered” or “new nurse*” or “new midwi*” or “new paramedic*” or “new* licensed” ) OR (MH "Novice Nurses") OR (MH "New Graduate Nurses") ) AND ( (MH "Work Engagement") or (MH "Personnel Turnover") OR ((work OR workplace OR job OR organi?ation* OR employee* OR occupation* OR profession*) N2 (engag* OR commit*)) OR ( intent* N2 (stay* OR leav*) ) OR turnover OR “personnel retention” ) AND ( (MH "Mentorship") OR (MH "Clinical Supervision") OR (MH "Preceptorship") or mentor* or precept* or “residency program*” OR “transition program*” ) ) AND ( review or meta-analysis or "meta synthesis" or meta-synthesis ) | 93 |
| OvidMedline | (new* qualified or new* graduate* or newcomer* or early career* or novice* or recent* graduate* or recent* registered or new nurse* or new midwi* or new paramedic* or new* licensed).ab,kf,ti. and (exp Work Engagement/ or exp Personnel Turnover/ or ((work or workplace or job or organi?ation* or employee* or occupation* or profession*) adj3 (engag* or commit*)).ab,kf,ti. or (intent* adj3 (stay* or leav*)).ab,kf,ti. or (turnover or personnel retention).ab,kf,ti.) and ((exp Mentoring/ or exp Preceptorship/ or (mentor* or precept* or residency program* or transition program* or clinical supervis*).ab,kf,ti.) and (review or meta-analysis or meta synthesis or meta-synthesis).ab,kf,ti.) | 56 |
| ProQuest | noft("new* qualified" OR "new* graduate*" OR newcomer* OR "early career*" OR novice* OR "recent* graduate*" OR "recent* registered" OR "new nurse*" OR "new midwi*" OR "new paramedic*" OR "new* licensed") AND noft(((work OR workplace OR job OR organi?ation* OR employee* OR occupation* OR profession*) NEAR/2 (engag* OR commit*)) OR (intent* NEAR/2 (stay* OR leav*)) OR turnover OR "personnel retention") AND noft("Clinical Supervis*" OR mentor* OR precept* OR "residency program*" OR "transition program*") AND noft(review or meta-analysis or "meta synthesis" or meta-synthesis ) AND review or meta-analysis or "meta synthesis" or meta-synthesis | 39 |
| Scopus | ( TITLE-ABS-KEY ( "new* qualified" OR "new* graduate*" OR newcomer* OR "early career*" OR novice* OR "recent* graduate*" OR "recent* registered" OR "new nurse*" OR "new midwi*" OR "new paramedic*" OR "new* licensed" ) AND TITLE-ABS-KEY ( ( ( work OR workplace OR job OR organi?ation* OR employee* OR occupation* OR profession* ) W/2 ( engag* OR commit* ) ) OR ( intent* W/2 ( stay* OR leav* ) ) OR turnover OR "personnel retention" ) AND TITLE-ABS-KEY ( "clinical supervis*" OR mentor* OR precept* OR "residency program*" OR "transition program*" ) AND TITLE-ABS-KEY ( review OR meta-analysis OR "meta synthesis" OR meta-synthesis ) ) | 77 |
| Cochrane | "new* qualified" or "new* graduate*" or newcomer* or "early career*" or novice* or "recent* graduate*" or “recent* registered” or “new nurse*” or “new midwi*” or “new paramedic*” or “new* licensed” [Work Engagement] or [Personel Turnover] or ((work OR workplace OR job OR organi?ation* OR employee* OR occupation* OR profession*) NEAR/2 (engag* OR commit*)) OR ( intent* NEAR/2 (stay* OR leav*) ) OR turnover OR “personnel retention”  AND "Clinical Supervis*" or mentor* or precept* or “residency program*” OR “transition program*” | 4 |
| Medic | mentor* perehdyt* työnohja* AND hoitaj* sairaanhoitaj* kätilö* ensihoitaj* terveydenhoitaj* | 181 |
| *Note.* MH= MESH -term; TIT/ti = title; ABS/ab. = abstract; KEY/kf = key words. | | |
