# Supplementary material for: THE Effect of Mentoring Programmes on Newly Graduated Nurses' Retention and Turnover: An Umbrella Review
Source: J Adv Nurs. 2025 Oct 27;82(7):6948–69. doi: 10.1111/jan.70326 (PMC13267445; doi:10.1111/jan.70326)
Supplement: Supplementary file 2 — Data S2: Assessment of methodological quality of included studies based on JBI‐QARI (Lockwood et al., 2020). [file JAN-82-6948-s002.docx]

Supplementary file 2. Assessment of methodological quality of included studies based on JBI-QARI (Lockwood et al., 2020)

| **Citation** | **Q1** | **Q2** | **Q3** | **Q4** | **Q5** | **Q6** | **Q7** | **Q8** | **Q9** | **Q10** | **Q11** | **Score** |
| --- | --- | --- | --- | --- | --- | --- | --- | --- | --- | --- | --- | --- |
| Ackerson & Stiles, 2018 | Y | Y | Y | Y | N | N | N | Y | Y | Y | Y | 8 |
| Asber, 2019 | Y | Y | Y | N | Y | N | N | N | Y | Y | Y | 7 |
| Brook et al., 2019 | Y | Y | Y | Y | Y | Y | N | Y | Y | Y | Y | 10 |
| Chen & Lou, 2014 | Y | Y | Y | Y | Y | Y | N | Y | Y | Y | Y | 10 |
| Edwards et al., 2011 | Y | Y | Y | Y | Y | Y | Y | Y | N | Y | Y | 10 |
| Edwards et al., 2015 | Y | Y | Y | Y | Y | Y | Y | Y | N | Y | Y | 10 |
| Ke et al., 2017 | Y | Y | Y | Y | Y | Y | Y | Y | Y | Y | Y | 11 |
| Rush et al., 2013 | Y | Y | Y | Y | Y | Y | N | Y | N | Y | Y | 9 |
| Rush et al., 2019 | Y | Y | Y | Y | Y | N | N | Y | N | Y | Y | 8 |
| Salt et al., 2008 | Y | Y | N | Y | Y | N | N | Y | Y | Y | Y | 8 |
| Vázquez‐Calatayud & Eseverri‐Azcoiti, 2023 | Y | Y | Y | Y | Y | Y | Y | Y | Y | Y | Y | 11 |
| Vidal & Olley, 2021 | Y | N | N | Y | Y | N | N | Y | N | Y | Y | 6 |
| Zhang et al., 2016 | Y | Y | Y | Y | Y | Y | Y | Y | Y | Y | Y | 11 |
| Total (%) | 100 | 92 | 85 | 92 | 92 | 62 | 38 | 92 | 62 | 100 | 100 |  |

Y = yes, N = No, U = Unclear. JBI critical appraisal checklist for qualitative research: Q1 = Is the review question clearly and explicitly stated? Q2= Were the inclusion criteria appropriate for the review question? Q3 = Was the search strategy appropriate? Q4 = Were the sources and resources used to search for studies adequate? Q5 = Were the criteria for appraising studies appropriate? Q6 = Was critical appraisal conducted by two or more reviewers independently? Q7 = Were there methods to minimize errors in data extraction? Q8 = Were the methods used to combine studies appropriate? Q9 = Was the likelihood of publication bias assessed? Q10 = Were recommendations for policy and/or practice supported by the reported data? Q11 = Were the specific directives for new research appropriate?
